# Biped Walking Based on Stiffness Optimization and Hierarchical Quadratic Programming

**DOI:** 10.3390/s21051696

**Published:** 2021-03-02

**Authors:** Xuanyang Shi, Junyao Gao, Yizhou Lu, Dingkui Tian, Yi Liu

**Affiliations:** 1 School of Mechatronical Engineering, Intelligent Robotics Institute, Beijing Institute of Technology, Beijing 100081, China; shixuanyang@bit.edu.cn (X.S.); 3120180169@bit.edu.cn (Y.L.); tiandingkui@bit.edu.cn (D.T.); 2 Beijing Advanced Innovation Center for Intelligent Robots and Systems, Beijing 100081, China; 3 Beijing Institute of Astronautical Systems Engineering, Beijing 100076, China; yiliu@bit.edu.cn

**Keywords:** spring-loaded inverted pendulum (SLIP), stiffness optimization, uneven ground walking, hierarchical quadratic optimization

## Abstract

The spring-loaded inverted pendulum model is similar to human walking in terms of the center of mass (CoM) trajectory and the ground reaction force. It is thus widely used in humanoid robot motion planning. A method that uses a velocity feedback controller to adjust the landing point of a robot leg is inaccurate in the presence of disturbances and a nonlinear optimization method with multiple variables is complicated and thus unsuitable for real-time control. In this paper, to achieve real-time optimization, a CoM-velocity feedback controller is used to calculate the virtual landing point. We construct a touchdown return map based on a virtual landing point and use nonlinear least squares to optimize spring stiffness. For robot whole-body control, hierarchical quadratic programming optimization is used to achieve strict task priority. The dynamic equation is given the highest priority and inverse dynamics are directly used to solve it, reducing the number of optimizations. Simulation and experimental results show that a force-controlled biped robot with the proposed method can stably walk on unknown uneven ground with a maximum obstacle height of 5 cm. The robot can recover from a 5 Nm disturbance during walking without falling.

## 1. Introduction

A spring-loaded inverted pendulum (SLIP) can be used to predict the spatial trajectory of the center of mass (CoM), energy wave, and ground reaction force during human walking, running, jumping, and other motions [1]. Many animal and human motion experiments have confirmed the good predictive performance of the SLIP model [2,3]. Geyer et al. determined the relationship among the landing angle, spring stiffness, and total energy of the SLIP model [4]. The SLIP model has unique physical characteristics and is thus convenient for generating real-time trajectories. The CoM velocity can be controlled by simply adjusting the landing point or touchdown angle. Raibert calculated foot placement based on a CoM-velocity feedback controller to adjust the trajectory and developed a legged robot with a running gait, with evaluation conducted in a simulation and a physical environment [5]. RHex, ATRIAS, and Minitaur are designed with physics elastic leg and direct drive joint to realize low inertia leg and torque control joint. The SLIP model is used to design the leg motion strategy, and realized uneven ground movement of the physical robot [6,7,8]. Compared with the linear inverted pendulum model, the elastic motion characteristics of the SLIP model allow it to greatly reduce the complexity of trajectory planning. The SLIP model has many advantages for simulating biological gait, real-time trajectory planning, and disturbance rejection. The flight phase in running gait is relatively simple, so the corresponding technology is relatively mature. In contrast, there are still some problems regarding walking gait, especially for walking on unknown uneven ground.

For walking gait, Rummel et al. analyzed the virtual spring stiffness range in stable walking with a double-support phase [9]. To support leg length contraction during walking, Rezazadeh et al. designed a leg extension and contraction control strategy combined with velocity decoupling control to achieve high-speed walking for the ATRIAS robot [10]. Schwind et al. improved the feedback control rate of the forward speed in a decoupling controller based on a stability analysis of the bouncing fixed point [11]. Andrews et al., Clark et al., and Schmitt et al. designed a linear update method that uses the current touchdown angle, flying angle, and target touchdown angle to improve the stability, balance, and self-recovery ability of the SLIP system on uneven ground [12,13,14]. Wu et al. extended the two-dimensional inverted pendulum to three dimensions and proposed a time-based deadbeat controller to achieve disturbance recovery and error tracking [15].

Previous research has already shown that a variable stiffness in the support leg can help in controlling energy of the SLIP. Jafarian et al. analyzed the walking performance with different spring stiffness [16]. Ghorbani et al. proposed an event-based time-independent controller, based on geometric progression with exponential decay in the kinetic energy error, which adjusts the stiffness of the support leg spring to control the injected energy to the biped in tracking a desired speed at the midstance [17]. However, this method can only effectively control the total energy, and it is difficult to accurately control the speed and height of the robot in all directions. Wensing et al. constructed an apex return map combined with human body motion cadence and standing time data, used nonlinear optimization to get different spring stiffness and touchdown angle offline, and used a first-order approximate deadbeat controller to achieve trajectory tracking to achieve high-speed periodic step running [18]. Liu et al. added ground height information and a double-support phase and extended the above method to uneven ground walking; however, the optimization process was still offline [19]. The method based on stiffness optimization of the SLIP mainly faces three problems. Firstly, there is no flight phase in the walking process, the swing leg cannot move to the desired foot placement in advance during the flight phase, and the swing leg cannot land with the slack length, so it is difficult to estimate the next touch down state. Secondly, since there is no flight phase, the apex state cannot be selected as the discrete state of regression mapping, the reselected state should include the state after landing and have enough time for the swing leg to move. Thirdly, the optimization method has a long computational time, so it needs to be accelerated to achieve the calculation within a control period.

The SLIP model provides only the CoM trajectory of the simplified model. Therefore, a lot of studies perform control mapping from the simplified model to the high-dimensional robot model. Khatib used the inverse dynamics method to realize mapping from the operation space control to the joint space [20]. The whole-body inverse dynamics method has become popular in humanoid robot control. Hutter et al. treated robot floating-based inverse dynamics as a quadratic programming (QP) problem and provided a method for solving the constraints [21]. Feng et al. applied whole-body inverse kinematics QP optimization based on whole-body inverse dynamics and realized robot force-position control [22]. Dai et al. considered momentum and angular momentum as the control targets and used QP to solve the central dynamics tracking problem [23]. However, the numbers of QP control targets and optimization variables are large, making the computational cost high. In addition, with the traditional QP method, it is difficult to guarantee absolute priority because tasks have different importance levels. Kanoun et al. decomposed the original QP problem into a sequence of QP problems according to priority to ensure absolute priority [24]. Inspired by [24], the present study improves hierarchical quadratic programming (HQP). The robot dynamics equation is rewritten as an optimization goal and given the highest priority. This eliminates the equality constraints for solving a sequence of QP problems.

Therefore, this paper mainly improves in these aspects, which are also the main contributions of this paper.

(1) The velocity feedback controller was used to calculate the foot placement in every control period, which is respectively used to predict the next landing state and generate the actual trajectory of the swinging leg.
(2) The touch down state is taken as the regression mapping state, so that the spring stiffness can be updated immediately after the uneven ground disturbance.
(3) The optimization variables are reduced. Only the spring stiffness and damping coefficient are optimized to speed up the calculation. Meanwhile, the spring stiffness is divided into two parts based on midstance to increase the solving space.
(4) HQP is used to realize the mapping of the simple robot model to the whole-body dynamic model. Whole-body motion control is realized with a strict priority of tasks.

**Figure 1:**
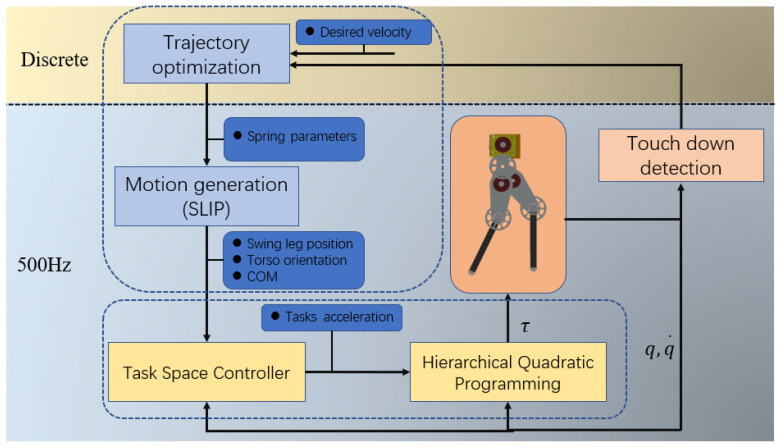
Block diagram of proposed optimization-based method. q  and $\dot{\;q\;}$ are the joint position and velocity, respectively, and τ is the joint torque.

A block diagram of the proposed control method is shown in Figure 1. The control system is divided into a discrete part and a continuous part. The discrete part is triggered by the swing leg touchdown and calculates the spring stiffness and damping coefficient through optimization. The continuous part control frequency is 500 Hz. Its main function is to achieve the desired trajectory generation, task space control, and whole-body motion control.

## 2. Trajectory Optimization of Center of Mass

### 2.1. Spring-Loaded Inverted Pendulum Model

**Figure 2:**
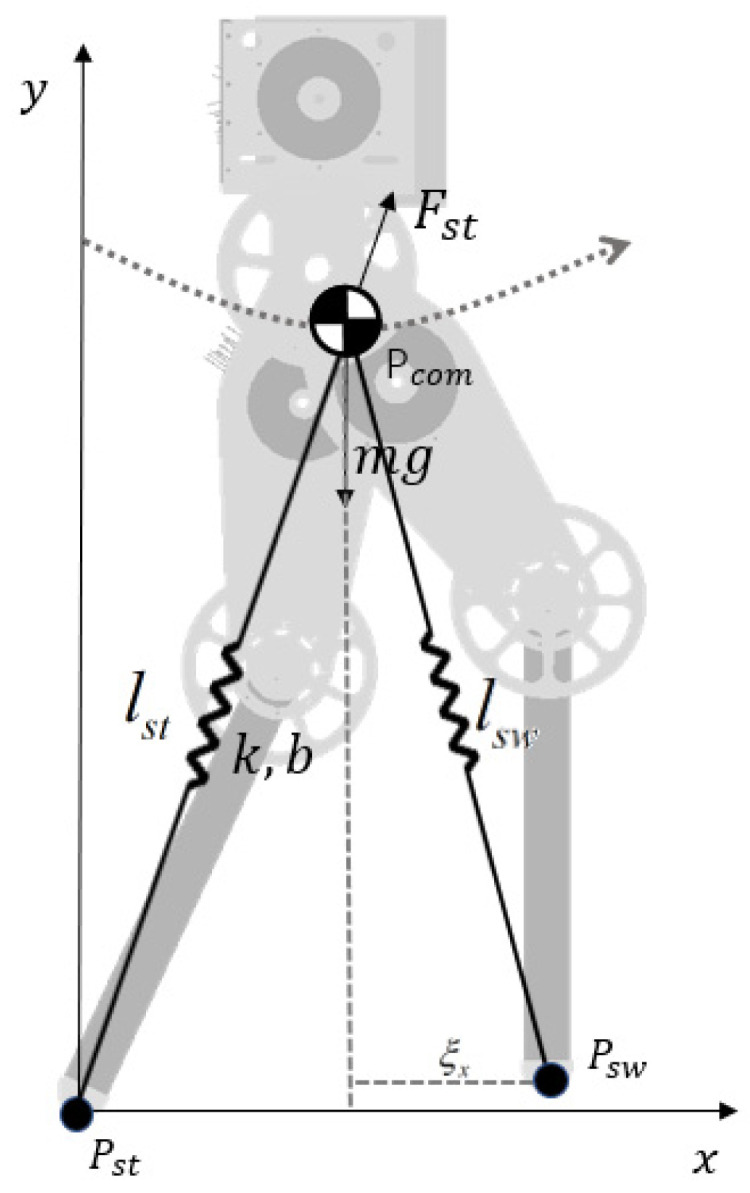
Spring-loaded inverted pendulum (SLIP) model of a humanoid robot.

The SLIP model consists of a mass point and two massless virtual spring legs, as shown in Figure 2. For each step during walking, the legs are divided into the support leg and the swing leg. The coordinates of the ends of the support and swing legs are $\;P_{st}=\left(x_{st},y_{y_{st}}\right)$ and $P_{sw}=\left(x_{sw},y_{sw}\right)$, respectively. The CoM coordinates are $P_{com}=\left(x_{\mathrm{com}},y_{com}\right)$. The world coordinates are fixed to the support foot. In the equations below, *k* is the virtual spring stiffness, *b* is the damping coefficient, $l_{slack}$ is the slack length of the spring, and $l_{st}$ is the actual length of the stance leg.

Since it is difficult to obtain an analytical solution for the asymmetric SLIP system, numerical integration was used to describe the CoM motion.

(1) $$l_{st}=\sqrt{x_{com}{\left(i\right)}^{2}+y_{com}{\left(i\right)}^{2}}$$

(2) $$\left[\begin{array}{c} F_{x}(i)\\ F_{y}(i) \end{array}\right]=\left(k*\left(l_{slack}-l_{st}\right)+b*\left({\dot{l}}_{slack}-{\dot{l}}_{st}\right)\right)\cdot \left[\begin{array}{c} x_{com}(i)\\ y_{com}(i) \end{array}\right]\cdot \frac{1}{l_{st}}$$

(3) $$\left[\begin{array}{c} ddx_{com}(i)\\ ddy_{com}(i) \end{array}\right]=\frac{1}{m}\left[\begin{array}{c} F_{x}(i)\\ F_{y}(i) \end{array}\right]+\left[\begin{array}{c} 0\\ -g \end{array}\right]$$

### 2.2. Touchdown Return Map

**Figure 3:**
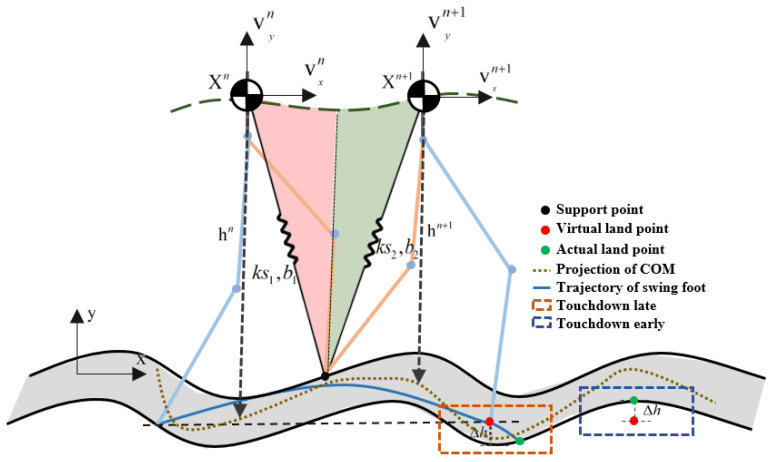
Touchdown return map of one step.

Our goal was to control the SLIP model from one step to the next by varying foot placement and spring characteristics. The walking process can be simplified by defining a Poincaré map to convert the system into a series of discrete states. Raibert [5] used the apex state before touchdown as the control state variable whereas Vejdani [25] used the vertical leg orientation state in the mid support period. For both of these methods, it is difficult to model the state changes caused by uneven ground. Therefore, the CoM state during touchdown was used as the control variable in the present study, as shown in Figure 3.

The robot state variables are the CoM velocity in two directions and the height in the vertical direction. The state variable of the Poincaré map is defined as $X^{n}={\left[v_{x}^{n},v_{y}^{n},h^{n}\right]}^{T\;}$ in step n and $X^{n+1}={\left[v_{x}^{n+1},v_{y}^{n+1},h^{n+1}\right]}^{T}\;$ in step *n* + 1. The virtual spring stiffness and damping are divided into two parts according to the midstance, which is based on an estimate of the duration of the support period. The input vector is selected as $u={\left[ks_{1},ks_{2},b_{1},b_{2}\right]}^{T}$, where $\;ks_{1}\;\mathrm{and}\;ks_{2}$ are the spring stiffness and $b_{1}\;\mathrm{and}\;b_{2}$ are the spring damping coefficient. The discrete return map can be expressed as

(4) $$X^{n+1}=f\left(X^{n},u\right)$$

To reduce optimization complexity, foot placement is not used as an optimization variable; instead, it is directly generated by the CoM feedback controller. In the equation below, $\xi_{x}$ represents the desired distance at touchdown between the swing foot and CoM in the x direction. $V_{x}$ and $V_{x}{}^{d}$ are the actual velocity and desired velocity in the x direction, respectively, and $\int {V_{x}}^{n+1}\mathrm{dt}$ and $\int {V_{x}}^{a}\mathrm{dt}$ are the desired position and actual position of the CoM relative to the world coordinate system in the x direction, respectively. $K_{vx}$, $K_{px}$, and $K_{Ix}$ are the velocity feedforward coefficient, velocity feedback coefficient, and position feedback coefficient, respectively. Note that this equation was applied during the whole support phase and that the result for each control period is used as the expected foot placement in the period.

(5) $$\xi_{x}=K_{vx}\cdot V_{x}{}^{a}+K_{px}\left(V_{x}{}^{a}-V_{x}{}^{n+1}\right)+K_{Ix}\left(\int V_{x}{}^{a}\mathrm{dt}-\int V_{x}{}^{n+1}\mathrm{dt}\right)$$

To predict the footing point and pass over obstacles, we set the swing leg length. $T_{sw}$ is defined as the estimated time of one step, $l_{ret}$ is defined as the maximum contraction of the swing leg, and $l_{slack}$ is defined as the final length. The support leg length at the last moment of the previous step was used as the initial length of the swing leg. The length of the swing leg during one step can be obtained via sixth-order interpolation. Given that the ground height was unknown, the next step landing height could not be obtained in the prediction process. Here, we define virtual foot placement, for which it is assumed that the height is the same as that in the previous step, as shown in Figure 3. When the swing foot height is the same as the virtual foot height, the robot has reached step *n* + 1.

(6) $$y_{sw}=y_{st}$$

### 2.3. Stiffness Optimization

Given an initial state $X^{n}$ at the current touchdown time and the desired state of the next touchdown time, the mapping relationship of f can be obtained using Equations (1)–(6). The optimized form of the problem can be expressed as Equation (7). An additional inequality constraint was added to ensure that the robot leg length does not exceed the slack length during the solution process (i.e., the robot does not have a flying phase). The optimization equation can be solved within 2 ms using the lsqnonlin function in MATLAB.

(7) $$\begin{array}{l} \underset{u}{min}\|X_{d}^{n+1}-f{\left(X^{n},u)\right\|}^{2}\\ \begin{array}{cc} st. & l< \end{array}l_{slack} \end{array}$$

### 2.4. Real-Time Trajectory Generation

Using Equation (7), we calculated the spring stiffness and damping coefficient required to reach the desired state. In the actual walking process of the step support phase after the robot touched the ground, we used Equations (6)–(8) to generate the reference CoM trajectory of the current step based on the updated spring parameters in each control cycle. The foot placement of the swing leg was obtained using Equation (5) according to the actual velocity, which allows the foot placement to be adjusted in real time. When the virtual landing point is lower than the actual landing point, the swing leg touches down early and the robot switches to the next step. When the virtual landing point is higher than the actual landing point, the swing leg touches down late and the robot continues to move. In this period of time, the current spring parameters were used to generate the robot CoM trajectory. The state error generated at this stage is controlled in the next step.

**Figure 4:**
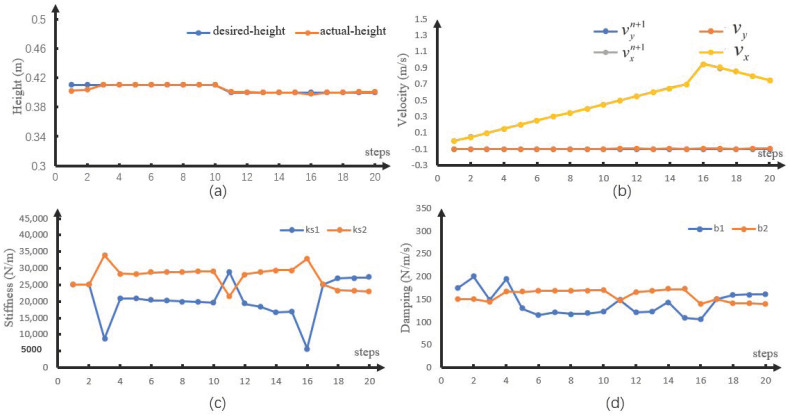
Numerical simulation results. (**a**) Discrete height, (**b**) discrete velocity, (**c**) discrete spring stiffness, and (**d**) discrete damping coefficient.

To verify the proposed method, we used the SLIP model to perform a numerical simulation in MATLAB. The simulation was running in the computer with Intel(R) Xeon(R) W-2145 CPU@3.70GHz. The parameters were m=10 kg and $l_{slack}=0.5\;m$. The control period was 2 ms. The robot walked a total of 20 steps in the simulation. To verify performance at different heights and velocities, the height of the CoM in the first 10 steps was set to 0.41 m and that in the last 10 steps was set to 0.40 m. Figure 4a,b shows that the model can well track the desired velocity and height. Figure 4c,d shows the spring stiffness and damping coefficients optimized during each step of the walking process. When the robot accelerated, the stiffness value of the virtual spring $ks_{1}$ was less than that of the spring $ks_{2}$. This is because the spring compression was the same before and after vertical leg orientation and the energy of the system was increased by the increase in spring stiffness, resulting in acceleration. When the robot decelerated, the situation was reversed.

## 3. Whole Body Control Based on Hierarchical Quadratic Programming

Whole-body robot control based on QP and weights has two problems. One is that the robot joints have many degrees of freedom (DoFs) and the task space is large, resulting in complicated calculation for QP optimization. The other is that it is difficult to achieve absolute priority between different tasks using weights to assign task priorities. Therefore, HQP is used to achieve fast and priority-based control by gradually reducing the solution space and solving a series of QP problems according to priority.

### 3.1. Hierarchical Quadratic Programming

HQP optimization is usually used to solve whole-body dynamics control. A linear cost function can be transformed into a standard quadratic programming problem [20]. Here, the form of the optimization function is modified. First, equality constraints are added to the cost function. Second, a slack factor is added to the inequality constraints to ensure that each optimization has a solution. The optimization form is as follows:

(8) $$\begin{array}{l} \begin{array}{cc} \underset{v,X}{min} & \|A_{eq}X-b_{eq}\|{}^{2}+{\left\|v\right\|}^{2} \end{array}\\ \begin{array}{cc} st. & A_{ueq}X-b_{ueq}\leq v \end{array} \end{array}$$

where X is the state variable to be solved and v is the slack factor. $A_{eq}^{2}=\left[A_{eq}^{1};\;A_{eq}^{2};\;\cdot \;;A_{eq}^{n}\right],\;\left[b_{eq}^{1};b_{eq}^{2};\;\cdot \;;b_{eq}^{n}\right],\;\left[A_{ueq}^{1};\;A_{ueq}^{2};\;\cdot \;;A_{ueq}^{m}\right],\;\mathrm{and}\;\left[b_{ueq}^{1};\;b_{ueq}^{2};\;\cdot \;;b_{ueq}^{m}\right]$ represent the coefficients of equality constraints and inequality constraints, *n* and *m* are the numbers of equality and inequality constraints, respectively. It should be noted that $A_{eq}^{i}$ could be the smallest unit of a task, such as the position of the CoM in the *x* direction, or a combination of multiple tasks, such as the position and posture of the swing foot. $A_{eq}^{1}$ to $A_{eq}^{n}$ are arranged in descending order of task priority.

For the highest-priority tasks $A_{eq}^{1}$ and $b_{eq}^{1}$, the following equations were solved using QP to obtain a solution $X_{1}^{*}$ that satisfies the highest-priority tasks, which can be obtained by solving the following equations with QP.

(9) $$\begin{array}{l} \begin{array}{cc} \underset{v,X_{1}^{*}}{min} & \|A_{eq}^{1}X_{1}^{*}-b_{eq}^{1}\|{}^{2}+{\left\|v\right\|}^{2} \end{array}\\ \begin{array}{cc} st. & A_{ueq}X_{1}^{*}-b_{ueq}\leq v \end{array} \end{array}$$

The solution of the (*i* + 1)-priority task is denoted as $X_{i+1}^{*}$; its value should not affect the result of higher-priority tasks.

(10) $$X_{i+1}^{*}=X_{i}^{*}+z_{i}\cdot u_{i+1}$$

The linear operator satisfies $\left[A_{eq}^{1};\;A_{eq}^{2};\;\cdot \;;A_{eq}^{i}\right]\;\cdot z_{i}=0$, so all $\;X_{i+1}^{*}$ possible solutions can satisfy the higher-priority optimization goal. Note that the optimization variables of the QP optimization at this priority are $u_{i+1}$ and $X_{i+1}^{*}$ is calculated using Equation (10).

(11) $$\begin{array}{l} \begin{array}{cc} \underset{v,u_{i+1}}{min} & \|A_{eq}^{i+1}\left(X_{i}^{*}+z_{i}\cdot u_{i+1}\right)-b_{eq}^{i+1}\|{}^{2}+{\left\|v\right\|}^{2} \end{array}\\ \begin{array}{cc} st. & A_{ueq}\left(X_{i}^{*}+z_{i}\cdot u_{i+1}\right)-b_{ueq}\leq v \end{array} \end{array}$$

The result $X_{n}^{*}$ obtained at the lowest priority is the final optimization result. The optimization problem is solved according to priority from high to low priority. A lower-priority solution is searched for in the null space of the higher-priority solution. As the priority decreases, the size of the null space decreases, and thus the solution process becomes faster.

### 3.2. Robot Configuration and Dynamics

**Figure 5:**
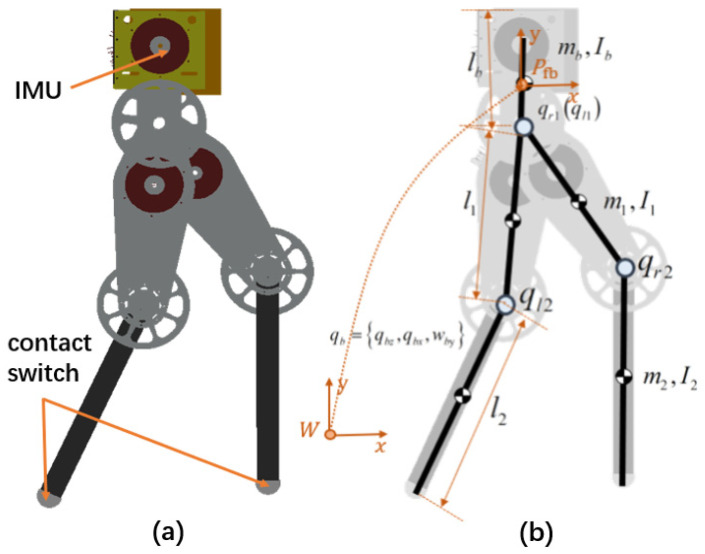
Force-controlled humanoid robot. An inertial measurement unit (IMU) is installed inside the chest and a contact switch is installed for each foot. Each leg has 2 degrees of freedom (DoFs). (**a**) Simulation model and (**b**) DoF configuration.

The DoF layout of the humanoid robot used in the present study is shown in Figure 5. There are seven DoFs in total, including two DoFs for each leg and three floating DoFs. An inertial measurement unit (IMU) is installed on the upper body of the robot to measure its posture and acceleration. The robot is point-footed. A pressure switch is installed at the end of the foot to detect the contact state. The floating coordinate system is established and fixed to the waist of the robot. $q_{b}=\left\{q_{bz},q_{bx},w_{by}\right\}$ is the generalized joint variable of the floating base; it represents the position and Euler angle of the floating base viewed in the world coordinate frame. $q_{j}=\left\{q_{r1},q_{r2},q_{l1},q_{l2}\right\}$ are the generalized coordinates of the active joints of the right and left legs. The robot’s active joints and floating joints are denoted as its generalized joint coordinate variable $q=\left\{q_{b},q_{j}\right\}$, which is a 7 × 1 vector. Each link of the robot has mass $m=\left\{m_{b},m_{thigh},m_{shank},m_{thigh},m_{shank}\right\}$ and inertia $I=\left\{I_{b},I_{thigh},I_{shank},I_{thigh},I_{shank}\right\}$.

The dynamics of robot can be expressed as

(12) $$\{\begin{array}{c} D\left(q\right)\overset{\cdot \cdot }{q}+h\left(q,\overset{\cdot }{q}\right)=B\tau +J^{T}\left(q\right)F\\ J\left(q\right)\overset{\cdot \cdot }{q}+\overset{\cdot }{J}\left(q\right)\overset{\cdot }{q}=0 \end{array}$$

where $\tau \in {\mathbb{R}}^{7\times 1}$ is the torque of the generalized joint and $F\in {\mathbb{R}}^{4\times 1}$ is the contact force and torque of the two feet. The contact force of the swing leg is set to zero during the single-support phase. $D\left(q\right)\in {\mathbb{R}}^{7\times 7}$ is the inertia matrix, $B\in {ℜ}^{18\times 18}$ is the joint torque mapping matrix, and $J^{T}\left(q\right)\in {\mathbb{R}}^{7\times 4}$ is the Jacobian matrix of the contact point. $h\left(q,\overset{\cdot }{q}\right)\in {\mathbb{R}}^{7\times 1}$ is the matrix of the centrifugal force, Coriolis force, and gravity.

### 3.3. Task Space Controller and Constraint

At the kinematics level, the walking tasks of the humanoid robot are mainly divided into the following control tasks: the position of the CoM, the position of the swing leg, and the upper body posture. At this level, there is no need to consider the dynamics of the robot. For the above tasks, a proportional-derivative feedback and feedforward controller was used to get the acceleration control values, as shown in Equation (13), where $P_{d}=\left\{p_{com},p_{swing},p_{posture}\right\}$ represents the desired position of the task space CoM, swing legs, and upper body posture, *P* represents the actual position, and $\overset{\cdot \cdot }{P_{d}^{*}}$ represents the output acceleration of the task space controller.

(13) $$\overset{\cdot \cdot }{P_{d}^{*}}=k_{p}\left(P_{d}-P\right)+k_{d}\left(\overset{\cdot }{P_{d}}-\overset{\cdot }{P}\right)+\overset{\cdot \cdot }{P_{d}}$$

Task *P* is differentiated twice to get the mapping from the task space acceleration to the joint space acceleration. *J_P_*(*q*) is the Jacobian of *P*.

(14) $$J_{P}(q)\overset{\cdot \cdot }{q}=\overset{\cdot \cdot }{P_{d}^{*}}-\overset{\cdot }{J_{P}}(q)\overset{\cdot }{q}$$

To satisfy the dynamic characteristics of the interaction between the robot and the actual physical environment during the walking process, the optimization solutions need to be limited. For the robot in the present study, the ground contact torque was set to 0 in the dynamic modeling stage, so there is no need to consider the pressure center constraint. Joint torque output and friction constraints were added to the optimization solution.

(15) $$\{\begin{array}{c} \tau_{min}\leq \tau \leq \tau_{max}\\ -uF_{x}\leq F_{x}\leq uF_{z} \end{array}$$

### 3.4. Prioritized Whole-Body Control

To avoid the construction of a contact dynamics model and direct control of the contact force, the state variables are written as $X={\left[\overset{\cdot \cdot }{q},\tau ,F\right]}^{T}\in {\mathbb{R}}^{18\times 1}$. For whole-body robot control, the robot dynamics equality constraints and the task space control objectives were included in the optimization cost function. Since the equality constraints of dynamic equation are the properties of the robot, it is necessary to give dynamics-related items the highest priority.

The ground contact constraint is given the second-highest priority. For the task space target, according to the impact on the walking task, the priority is given to CoM tracking, swing leg tracking, and upper body posture tracking. The optimization coefficient matrix is shown in Table 1.

The solution $X_{n}^{*}$ can be obtained using Equations (8)–(11). Note that the problem in the solution space of the highest priority can be solved without using QP. With the state variable containing τ and F, it can be reasonably assumed that τ=0 and F=0. These two variables satisfy the inequality constraints. By substituting them into the dynamic equation, we can obtain $X_{1}^{*}$ as

(16) $$X_{1}^{*}={\left[\overset{\cdot \cdot }{q},\tau ,F\right]}^{T}={\left[-D{\left(q\right)}^{-1}h\left(q,\overset{\cdot }{q}\right),0,0\right]}^{T}$$

The method used in this study more frequently calls QP optimization. However, the method used here has some advantages. First, each call of QP is used only for part of the optimization goal; low-priority tasks are searched for in the null space of higher-priority tasks and thus the search space is gradually reduced. The equality constraint is eliminated, so a single optimization is very fast. Second, for the highest priority, the results are directly obtained through inverse dynamics based on reasonable assumptions; that is, QP optimization is not used. Third, the final optimization results can strictly guarantee task priority. For each layer of QP optimization, we can solve the optimization problem using MATLAB’s QP solver quadprog. The HQP problem can be solved within 0.05 ms.

## 4. Simulation and Experiment

To demonstrate the proposed method for walking on unknown uneven ground, we performed a simulation and an experiment on a planar robot. The planar humanoid robot has a total mass of 4.87 kg. Table 2 shows the mass and inertia of the links in the model and lists the dimensions of the links of the robotic mechanism. The parameters of the left and right legs are the same, so only those for one leg are shown. The robot had 4 DoFs and each joint was driven by a brushless DC motor with a 5:1 gear ratio based on force control. A six-axis IMU (MPU6000) was mounted at the center of the upper body to measure the posture and acceleration. A pressure switch was installed on each foot to measure the contact state of the robot with the ground. The computer with Intel(R) Xeon(R) W-2145 CPU@3.70GHz was used as the robot computing platform.

### 4.1. Simulation

Simulink in MATLAB2018B and Adams 2018 software were used to verify the proposed methods. We built the robot simulation model in SolidWorks, including rigid bodies with mass and inertia and force-driven joints. A complex terrain composed of stairs, slopes, and irregular and rugged roads was constructed. A contact model between the robot and the road was added. The robot had two joints on each leg (four in total). A free-motion joint with three DoFs was added between the upper body and the ground. Its measured value was the output of the IMU. To make the simulation more realistic, Gaussian noise and a low-pass filter were added to the state values, including joint velocity, IMU data, and contact force. In the simulation, we used the force sensor to detect the contact state; the actual robot uses only a pressure switch. The coefficient of friction between synthetic rubber and concrete was 0.6–0.85, so the friction coefficient was set to 0.7. To verify the walking ability of the robot with the proposed method on complex terrain without the effect of driver limitation, the maximum joint torque of the robot in the simulation was set to be three times that of the actual robot.

**Figure 6:**
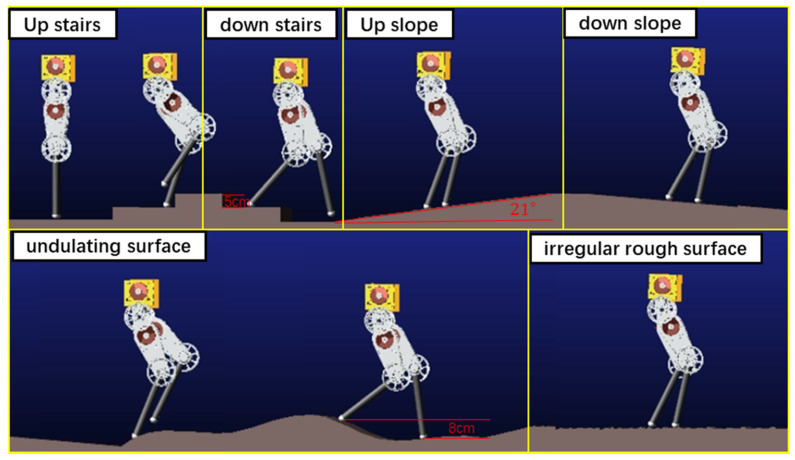
Robot walking on unknown uneven ground in simulation.

To verify the robot’s ability to walk on unknown uneven ground using the proposed method, we constructed a complex environment composed of multiple terrains in Adams 2018, as shown in Figure 6, including two steps going up and two steps going down (each 5 cm high), upward- and downward-sloping ramps with an incline angle of 21°, an undulating surface, and an irregular rough surface. The ratio of the maximum terrain height in one step to the robot leg length was 0.18. In the simulation process, the robot accelerated to 0.3 m/s in three steps, and then kept the velocity of 0.3 m/s to move forward. The desired height of the CoM was set as 0.40 m during support foot switching. The height of 0.45 m when the robot is standing was set as the slack length of the spring. In optimization, the initial spring stiffness and damping coefficient were set at 20,000 N/m and 100 Ns/m. The joint torque limit was set to 100 Nm. The CoM acceleration limitation was set as 10 m/s² and 8 m/s² in the x and y directions. Spring stiffness, damping, length, and CoM velocity were recorded during the walking process. The parameter values used in the simulation are shown in Table 3.

**Figure 7:**
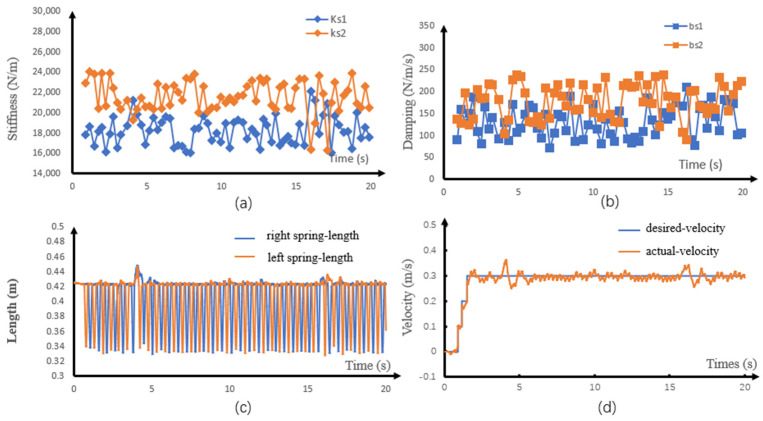
Simulation results of walking on unknown uneven ground. (**a**) Discrete stiffness, (**b**) discrete damping coefficient, (**c**) virtual spring length, and (**d**) center of mass (CoM) velocity.

**Figure 8:**
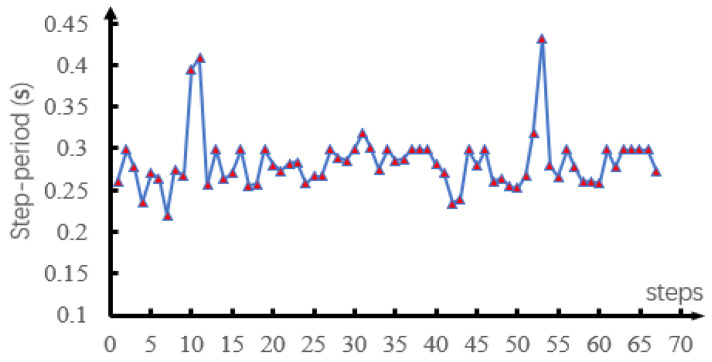
Step period of each step in simulation.

**Figure 9:**
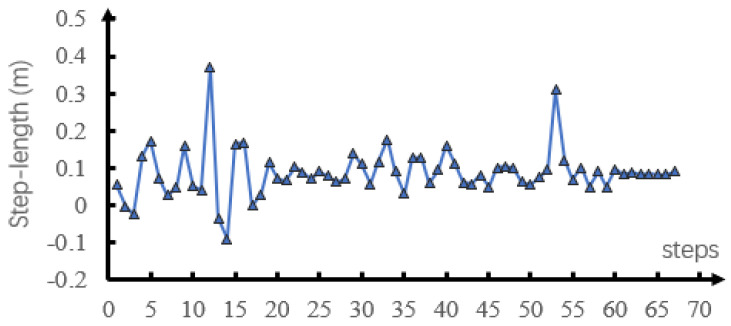
Step length of each step in simulation.

Figure 6 shows the walking process of the robot. The robot walked up the stairs at 1.8–3.0 s and down the stairs at 3.0–4.4 s. It walked up the slope at 4.4–8.2 s and down the slope at 8.2–12.7 s. Finally, it passed over the undulating surface at 12.7–17 s and the irregular rough surface at 17–20 s. Figure 7a,b respectively shows the spring stiffness and damping coefficient optimized in each step during support phase switching. The average spring stiffness was 18,187.6 and 21,555.8 N/m for ks1 and ks2, respectively, and the average damping coefficient was 129.5 and 178.2 Ns/m for b1 and b2, respectively. Figure 7c shows the actual length of the virtual spring during walking. During the whole process, the actual spring length was smaller than the slack length. Figure 8 and Figure 9 show the step period and step length of each step, respectively. Figure 7d shows that the maximum velocity error of the robot, which was caused by the unmodeled terrain, was 0.04 m/s. It was proved that our method could effectively control the CoM velocity of the robot and tracking the desired velocity.

For a point-footed robot, the walking process can simply be divided into flat walking, walking up, and walking down. In flat waking, the touchdown time, the foot placement, and the CoM state of the next touchdown moment are close to the expected value. The spring parameters and step size show periodicity. When walking down, because the virtual foot placement was higher than the actual foot placement, the robot needed to move until it touched the ground under the current parameters. For example, at 4.0 s, the robot was walking down the stairs, and thus the virtual spring length and velocity increased. With the difference in the foot placement height of the robot increased, the velocity error increased; When waking up, the swing leg will touchdown early compared to the desired trajectory, and immediately enter the next step. The COM accelerated in the second half of the midstance, so the swing leg touchdown early will cause the velocity to be lower than the expected velocity. When touchdown, due to the unknown terrain, the CoM cannot be controlled precisely to reach the desired state, but all errors can be accounted and controlled in the current step by reoptimization and will not accumulate in the next step. So, the robot can overcome the disturbance of uneven ground.

To verify the performance of the proposed method on robots with different sizes, we increased the robot height to 1.2 m. The ratio of the maximum terrain height in one step to the robot leg length was 0.096 and the robot mass was set to 65 kg. The spring model parameters were changed accordingly. This robot walked in the same environment. The simulation shows that the proposed method achieved better horizontal velocity control for a larger robot. The reason is that when the length of the virtual spring is longer, the spring compression is greater, and the ratio of the maximum terrain height to the spring compression is smaller, so the energy disturbance to the SLIP model is also smaller. Related videos can be viewed in the supporting material.

The proposed method achieves good control performance in the presence of an external force disturbance in the single-support phase. During the construction of the touchdown return map, we used Equation (5) to calculate the desired foot placement in each control cycle to optimize stiffness, and then calculated the desired CoM trajectory based on the spring stiffness. In the actual walking process, for the foot placement of the swing leg, the actual CoM velocity (not the desired CoM velocity) was used to calculate the actual foot placement. When there was no external force disturbance after the robot lands, ideally, the desired and actual CoM velocity of a single step should be consistent, and the desired and actual footing points should be consistent. When there is an external force disturbance, the CoM velocity will be disturbed, and the actual foot placement will be recalculated according to the actual velocity, so that the CoM velocity feedback controller retains the effect of real-time velocity control. Previous studies adjusted foot placement [17] using offline optimization and thus could not adjust it according to actual disturbances during the single-step support period. On flat ground, the robot with the proposed method walking at a velocity of 0.1 m/s can quickly recover the desired state without falling if an external force disturbance with a duration of 0.1 s and a total impulse of 5 Nm was applied during the single-step support period. Disturbance recovery was achieved via foot placement adjustment and whole-body dynamics control based on HQP. When the disturbance is small, the velocity can recover the desired state within the single-step support phase period via only whole-body dynamics control. When the disturbance is large, the control error of HQP is transmitted to the foot placement controller. Related videos can be viewed in the supporting material.

### 4.2. Experiment

**Figure 10:**
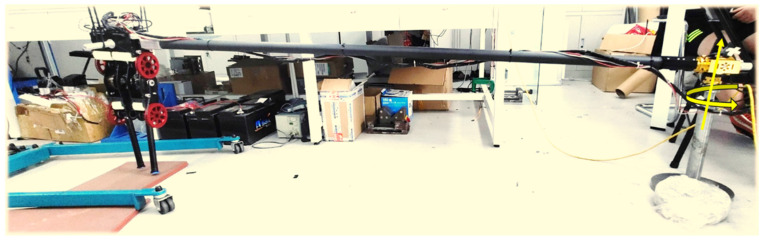
Experiment system.

We applied the proposed method to an actual robot for walking on unknown uneven ground. The joint configuration and structural parameters of the physical robot were consistent with the simulation model. Each joint consists of an external rotor motor with a maximum power of 400 W and a 5:1 synchronous belt, and was driven by an ELMO driver in the current control mode. NUC8i7HVK with intel Core i7-8809G CPU@3.10GHz was used as the control computer, and Windows-RTX was used as the real-time operating system of the robot. The six-axis IMU sensor of MPU6050 was installed on the upper body to measure the velocity and attitude. A push switch was installed on the foot to detect the contact state between the foot and the ground. The driver, IMU and switch was communicated with control computer by CANopen with a frequency of 500 Hz. Since the robot was planar, to avoid falling sideways, we designed a long arm mechanism with circular motion. The robot was fixed on the end of the arm and it could only move forward and backward or up and down in a circular trajectory. The circle radius was 2.5 m, as shown in Figure 10.

**Figure 11:**
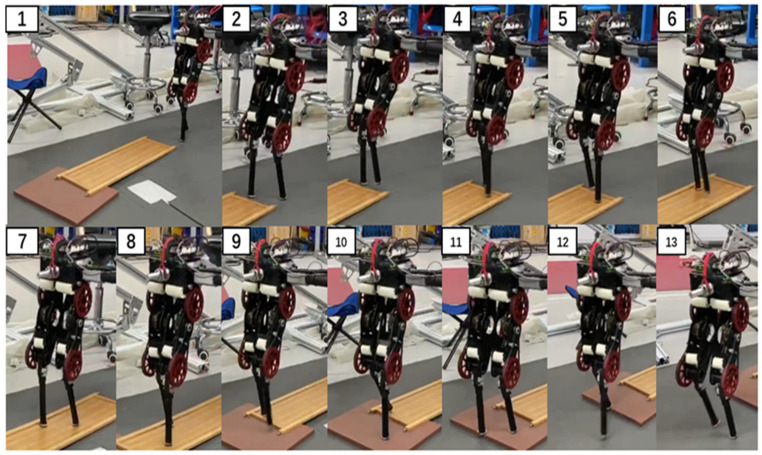
Photographs from the experiment of walking on unknown uneven ground. The desired velocity was set to 0.3 m/s and the desired height was set to 0.4 m.

**Figure 12:**
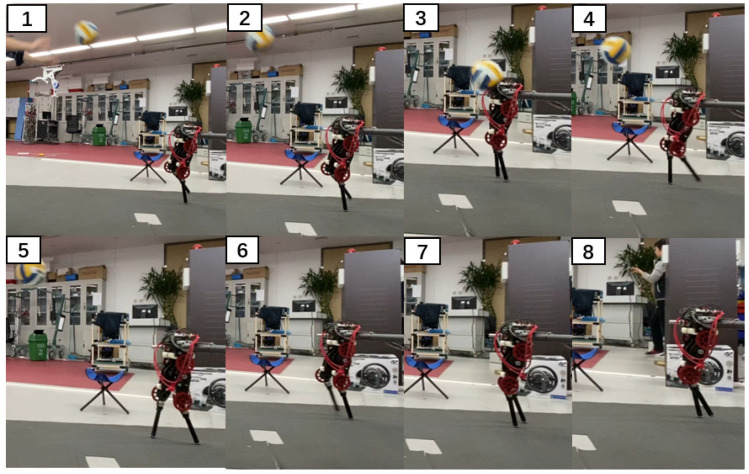
Photographs from the push recovery experiment. The front of the robot upper body was hit by a volleyball.

The push recovery performance was tested. Since the robot used in this experiment was two-dimensional, we used a rod that rotates around a fixed axis to limit its movement in the left and right directions. The robot could move along the circle at the end of the rod and in the up and down directions. During the experiment, the robot joint torque limit was set according to the real value. The whole-body controller used in the experiment was slightly modified compared with that used in the simulation because of differences between the actual and simulated robots. It is difficult to accurately respond to the desired torque command with a high frequency because the actual robot joint force control bandwidth and control accuracy were limited. Therefore, the optimization item $\min{\left\|\tau_{k+1}-\tau_{k}\right\|}^{2}$ for smooth output torque was added to the actual robot. This optimization item was given the lowest priority. The test environment included a 7° slope and 2-cm-high steps. Figure 11 shows the walking state of the robot moving over unknown uneven ground. Figure 12 shows the walking state of the robot after it was disturbed by a thrown volleyball. The robot showed good ability to traverse complex terrain and recover from external disturbances. The experiment can be viewed in the supplementary materials.

## 5. Discussion

In this paper, we applied the proposed method to a two-dimensional planar robot and showed good walking performance. We selected the state of touchdown moment as the regression mapping state variable, so that the optimization process can take the state disturbance at the moment of touchdown into consideration, so that the robot can control the disturbance and move the swinging leg to the desired foot placement at a complete walking step. The CoM velocity feedback controller was used to calculate the desired foot placement and used to estimate the touchdown state of the next step to construct the regression. The main reason that we do not take the foot placement as an optimization variable mainly for reducing the computational complexity and retaining the adjustment ability of the swing leg after the spring parameters are determined is to control the external force disturbance during the single-step support period. In addition, the spring stiffness in the single-step support period was divided into two parts by midstance, so that different stiffness could be applied in the compression stage and the elongation stage to control the system energy, which improved the optimization solution space. The main feature of the HQP method used here was the strict guarantee of task priority. In addition to tasks related to walking stability, it was important for robot operation tasks. For example, grasping tasks require high position/force control accuracy of the end of an arm in only a certain direction. The advantage of the method in this paper was that the robot could control the state in the step after touchdown, while the preview methods need to be controlled by adjusting the footing point or other parameters in the next step after touchdown. In the process of walking, the change of foot placement and stiffness was equivalent to replanning the trajectory and HQP used the whole-body instantaneous dynamics to track and control the trajectory. We only realized the real-time optimization of the planar robot, and it needed to consider the real-time calculation if extending the method to 3D. In addition, the spring stiffness was divided into two parts at midstance, where there will be a sudden change of the contact force, so the requirements of torque control of the physics robot were higher. These issues will be further discussed in future work.

## 6. Conclusions

We proposed a humanoid robot motion planning and control method based on spring stiffness optimization and HQP. Walking on unknown uneven ground and push recovery were demonstrated in a simulation and an experiment. The proposed method could be divided into two parts, namely discrete stiffness optimization and whole-body control. For real-time planning, a return map was constructed based on the touchdown moment and the spring stiffness was divided into two parameters based on the midstance. Foot placement was calculated using the velocity feedback controller (i.e., it was not an optimized variable). For whole-body control, the dynamic equations and contact constraints were rewritten as the optimization cost function. Tasks were prioritized according to importance, with tasks related to equality constraints given the highest priority. Sequential QP problems were solved in order of priority, with lower-priority QP problems solved in the higher-priority QP problem’s null space. A robot with the proposed method covered 5-cm-high stairs, 21° slopes, undulating ground with a maximum height of 6 cm, and irregular rough ground in a simulation. It recovered from an external force disturbance of 5 Nm. In an experiment, a real robot using the proposed method also displayed a good performance. The method presented in this paper achieved a good control performance in a planar robot. However, we can still make improvements in the following aspects. Adding more optimization goals, such as the contact force and foot placement, to generate a more human-like gait or using real data of human walking to generate robot walking trajectory through learning and improve the robot’s ability to perform tasks while walking [26,27].

## Figures and Tables

**Table 1:** Equality constraint coefficient matrix.

Priority	Description	$A_{eq}^{i}$	$b_{eq}^{i}$
1	Dynamic	$A_{eq}^{1}=\left[\begin{array}{ccc} D\left(q\right) & -B & -J^{T}\left(q\right) \end{array}\right]$,	$b_{eq}^{1}=-h\left(q,\overset{\cdot }{q}\right)$,
2	contact	$A_{eq}^{2}=\left[\begin{array}{ccc} J\left(q\right) & 0 & 0 \end{array}\right]$,	$b_{eq}^{2}=-\overset{\cdot }{J}\left(q\right)\overset{\cdot }{q}$,
3	CoM	$A_{eq}^{3}=\left[\begin{array}{ccc} J_{com}\left(q\right) & 0 & 0 \end{array}\right]$,	$b_{eq}^{3}=-{\overset{\cdot }{J}}_{com}\left(q\right)\overset{\cdot }{q}$,
4	Swing foot, posture	$A_{eq}^{4}=\left[\begin{array}{ccc} J_{swing}\left(q\right) & 0 & 0 \end{array}\right]$, $A_{eq}^{5}=\left[\begin{array}{ccc} J_{posture}\left(q\right) & 0 & 0 \end{array}\right]$,	$b_{eq}^{4}=-{\overset{\cdot }{J}}_{swing}\left(q\right)\overset{\cdot }{q}$, $b_{eq}^{5}=-{\overset{\cdot }{J}}_{posture}\left(q\right)\overset{\cdot }{q}$,

**Table 2:** Model parameters used in simulation.

Description	Value
$m_{b}$	2.088 kg
$m_{1}$	1.064 kg
$m_{2}$	0.329 kg
$I_{b}$	0.0044 kg·m²
$I_{1}$	0.0038 kg·m²
$I_{2}$	0.0028 kg·m²
$L_{b}$	0.152 m
$L_{1}$	0.25 m
$L_{2}$	0.25 m

**Table 3:** Weights and gains used in simulation.

Description	Value
$l_{slack}$	0.45 m
$T_{sw}$	0.3 s
[$K_{vx}$,$K_{px}$,$K_{Ix}$]	[0.12,0.2,0.1]
[$k_{p\_com}$,$k_{d\_com}$]	[[1000,700], [40,65]]
[$k_{p\_swing}$,$k_{d\_swing}$]	[[600,600], [35,35]]
[$k_{p\_posture}$,$k_{d\_posture}$]	[50,5.5]

## Data Availability

Data sharing not applicable.

## Supplementary Materials

The following are available online at https://www.mdpi.com/1424-8220/21/5/1696/s1. Videos have been submitted with the paper.
